# Epidemics of enterovirus infection in Chungnam Korea, 2008 and 2009

**DOI:** 10.1186/1743-422X-8-297

**Published:** 2011-06-13

**Authors:** KyoungAh Baek, SangGu Yeo, BaeckHee Lee, KwiSung Park, JaeHyoung Song, JeeSuk Yu, InSoo Rheem, JaeKyung Kim, SeoYeon Hwang, YoungJin Choi, DooSung Cheon, JoonSoo Park

**Affiliations:** 1 Department of Microbiology, Chungcheongnam-Do Institute of Health and Environmental Research, Daejeon, Korea; 2 Divison of Enteric and Hepatitis viruses, National Institute of Health, Korea Center for Disease Control and Prevention, Seoul, Korea; 3 JeongGene Pediatrics, Yeongi, Korea; 4 Department of Herbal Resources, Professional Graduate School of Oriental Medicine, Wonkwang University, Iksan, Korea; 5 Departments of Pediatrics, College of Medicine, Dankook University, Cheonan, Korea; 6 Departments of Laboratory Medicine, College of Medicine, Dankook University, Cheonan, Korea; 7 Department of Biology, College of Sciences, Kyunghee University, Seoul, Korea; 8 Departments of Laboratory Medicine, College of Medicine, Soonchunhyang University, Cheonan, Korea; 9 Departments of Pediatrics, College of Medicine, Soonchunhyang University, Cheonan, Korea

## Abstract

Previously, we explored the epidemic pattern and molecular characterization of enteroviruses isolated in Chungnam, Korea from 2005 to 2006. The present study extended these observations to 2008 and 2009. In this study, enteroviruses showed similar seasonal prevalent pattern from summer to fall and age distribution to previous investigation. The most prevalent month was July: 42.9% in 2008 and 31.9% in 2009. The highest rate of enterovirus-positive samples occurred in children < 1-year-old-age. Enterovirus-positive samples were subjected to sequence determination of the VP1 region, which resolved the isolated enteroviruses into 10 types in 2008 (coxsackievirus A4, A16, B1, B3, echovirus 6, 7, 9, 11, 16, and 30) and 8 types in 2009 (coxsackievirus A2, A4, A5, A16, B1, B5, echovirus 11, and enterovirus 71). The most prevalent enterovirus serotype in 2008 and 2009 was echovirus 30 and coxsackievirus B1, respectively, whereas echovirus 18 and echovirus 5 were the most prevalent types in 2005 and 2006, respectively. Comparison of coxsackievirus B1 and B5 of prevalent enterovirus type in Korea in 2009 with reference strains of each same serotype were conducted to genetic analysis by a phylogenetic tree. The sequences of coxsackievirus B1 strains segregated into four distinct clusters (A, B, C, and D) with some temporal and regional sub-clustering. Most of Korean coxsackievirus B1 strains in 2008 and 2009 were in cluster D, while only "Kor08-CVB1-001CN" was cluster C. The coxsackievirus B5 strains segregated in five distinct genetic groups (clusters A-E) were supported by high bootstrap values. The Korean strains isolated in 2001 belonged to cluster D, whereas Korean strains isolated in 2005 and 2009 belonged to cluster E. Comparison of the VP1 amino acid sequences of the Korean coxsackievirus B5 isolates with reference strains revealed amino acid sequence substitutions at nine amino acid sequences (532, 562, 570, 571, 576-578, 582, 583, and 585).

## Introduction

Human enteroviruses (HEV) are RNA viruses from the picornaviridae family. Clinical consequences of HEV infections include the common cold, hand-foot-mouth disease, acute hemorrhagic conjunctivitis, myocarditis, encephalitis, poliomyelitis, and aseptic meningitis. The latter, which mainly affects young children, is the most commonly encountered illness associated with enteroviral infections, often appearing in the form of outbreaks [1-3]. More than 80 immunologically distinct serotypes cause infections in humans; they are grouped into polioviruses, echoviruses (ECV), coxsackievirus A (CVA), coxsackievirus (CVB), and enterovirus (EV) types 68-71. These viruses are also divided into several subgroups: polioviruses and HEV-A, HEV-B, HEV-C, and HEV-D [4-7].

Outbreaks of enteroviruses typically peak during the summer and early fall, and various serotypes are often associated with a single outbreak. The predominant enterovirus types vary from year-to-year, with ECV 9, 13, 18, and 30 and CVB 5 being the most frequently isolated in Europe and the United States over the past few years [8-10]. Since 1993, when nationwide surveillance began in Korea, there had been reports of summer outbreaks of enteroviruses, involving ECV 5, 6, 7, 9, 13, 18, and 30, CVA 24, CVB 3 and 5, and EV 71 [11,12].

The enterovirus genome contains a 7,500 nucleotide-long single-stranded RNA molecule with polarity. The 5' and 3' non-coding regions (NCRs) are generally highly conserved in general. The most variable regions of the genome are within the genes encoding the capsid proteins, VP1, VP2, VP3 and VP4, which are partially exposed on the virus surface [13-15]. Laboratory diagnosis of enterovirus infections is based on amplification of highly conserved regions within the enteroviral RNA genome, The 5'NCR seems to be the most conserved region among enteroviruses and is therefore targeted widely in diagnostic procedures [16,17]. In addition to traditional virological methods used to identify the enterovirus serotype, reverse transcription-polymerase chain reaction (RT-PCR) methods based on amplification of VP1 region have been recently developed [18-20]. Since the VP1 region is one of the main exposed regions of the viral capsid and has been suggested to include a serotype specific antigenic neutralization site, the BC loop in the VP1 region is, in particular, one of the regions associated with viral antigenicity, and substitutions resulting in conformational changes in this region are believed to play a role in host adaptation for enteroviruses [21-23]. Appropriately, the partial VP1 sequences were compared with a database of complete enterovirus VP1 sequences of all serotypes to determine whether the isolates were related genetically to any known enterovirus serotype [19]. In addition, phylogenetic analysis from sequence data of the VP1 region is considered to be a standard method of molecular analysis for epidemiological purposes and the clustering or genotyping in combination with phylogenetic analysis is able to discriminate between lineages within a serotype and to identify emerging new variants or serotypes [24].

An epidemic due to enterovirus occurred in Chungnam, Korea in 2008 and 2009. Presently, to determine the enterovirus serotypes of the epidemic, viral cultures were carried out by inoculating samples to susceptible cell lines and examining the cytopathic effects. Also, molecular detection was performed using 5' NCR RT-PCR and sequencing of the VP1 region of enteroviruses. The aim was to determine the epidemiology of the enterovirus infection and molecular characteristics of the Korean CVB 1 and CVB 5 isolates.

## Materials and methods

### Virus isolation

Using susceptible cell lines such as rhabdomyosarcoma (Rd), Vero, and buffalo green monkey (BGM) cells, enteroviruses were isolated from 1,214 clinical stool or cerebrospinal fluid (CSF) specimens from hospitalized patients whose symptoms were consistent with enterovirus infections in Chungnam, Korea in 2008 and 2009.

### RT-PCR

The testing algorithm for detection and molecular typing of enterovirus was previously described [11]. Cells exhibiting 70% cytopathic effects were frozen and thawed three times and viral RNA was extracted from the supernatant of the infected cells using Magnetic-beads (Toyobo, Osaka, Japan). The extracted RNA was dissolved in 50 μL of nuclease-free water and stored at -70°C until used for RT-PCR. For cDNA synthesis, a 20 μL reaction mixture containing 5 μL of each viral RNA, 0.2 μL primer (AN32, AN33, AN34, and AN35) (Table 1), 4 μL of 5X reverse transcriptase buffer, 2 μL of 0.1 M dithiothreitol (DTT), 4 μL of 10 mM M-MLV reverse transcriptase (Invitrogen, Carlsbad, CA) was used. The mixture was reacted at 20°C for 10 min, 37°C for 120 min, 95°C for 5 min, and then chilled on ice. PCR was performed using a primer set specific for the 5' NCR of the enterovirus as described previously [25]. Briefly, a 50 μL reaction mix containing 0.2 μM of primers ENT-F and ENT-R (Table 1), 2U of *Taq *DNA polymerase (Promega, Madison, WI), 100 μM concentrations of mixture of dNTPs, and 2 μM MgCl_2 _was amplified by 35 cycles of 94°C for 1 min, 52°C for 1 min, and 72°C for 1 min. The final extension step was extended to 72°C for 7 min. Semi-nested PCR amplifying the VP1 coding region was carried out as described previously [18]. In the initial PCR, a 50 μL reaction mix containing 0.2 μM of primers 224 and 222 (Table 1), 2 U of Taq DNA polymerase (Promega), 100 μM concentrations of mixture of dNTPs, a 2 μM MgCl_2 _was amplified by 40 cycles of 95°C for 30 sec, 42°C for 30 sec, and 60°C for 45 sec. One microliter of the first PCR product was added to a second PCR for semi-nested amplification. Fifty microliters of a reaction mix containing 0.2 μM of primers AN89 and AN88 (Table 1), 2.5 U of Taq DNA polymerase (Promega), 100 μM concentrations of a mixture of dNTPs, and 2 μM MgCl_2 _was incubated at 95°C for 6 min prior to 40 amplification cycles of 95°C for 30 sec, 60°C for 20 sec, and 72°C for 15 sec.

**Table 1:** Candidate enteroviruses isolated in this study

Isolate	Diagnosis	Gender	Age	Month of Isolation	Specimen	Accession no.	Type
Kor08-CVB1-001cn	Aseptic meningitis	M	1	Jun. 2008	Stool	HQ685862	Coxsackievirus B1
Kor08-CVB1-015cn	Aseptic meningitis	M	2	Jul. 2008	CSF/Stool	HQ685863	
Kor08-CVB1-060cn	Urinary tract infection	M	0	Aug. 2008	CSF/Stool	HQ685864	
Kor09-CVB1-008cn	Herpangina	M	1	Jun. 2009	Stool	HQ685865	
Kor09-CVB1-041cn	Septicaemia	F	0	Jun. 2009	Stool	HQ685866	
Kor09-CVB1-052cn	Septicaemia	M	0	Jun. 2009	Stool	HQ685867	
Kor09-CVB1-069cn	Aseptic meningitis	F	0	Jul. 2009	Stool	HQ685868	
Kor09-CVB1-078cn	Aseptic meningitis	F	0	Jul. 2009	Stool	HQ685869	
Kor09-CVB1-085cn	Aseptic meningitis	M	0	Jul. 2009	Stool	HQ685870	
Kor09-CVB1-088cn	Septicaemia	M	0	Jul. 2009	Stool	HQ685871	
Kor09-CVB1-094cn	Aseptic meningitis	F	0	Jul. 2009	Stool	HQ685872	
Kor09-CVB1-095cn	Hand foot mouth disease	F	6	Jul. 2009	Stool	HQ685873	
Kor09-CVB1-110cn	Aseptic meningitis	M	3	Aug. 2009	Stool	HQ685874	
Kor09-CVB1-113cn	Aseptic meningitis	M	0	Aug. 2009	Stool	HQ685875	
Kor09-CVB1-115cn	Acute tonsillitis	M	0	Aug. 2009	Stool	HQ685876	
Kor09-CVB1-118cn	Acute pharyngotonsillitis	M	3	Aug. 2009	Stool	HQ685877	
Kor09-CVB1-130cn	Aseptic meningitis	F	0	Sep. 2009	Stool	HQ685878	

Kor09-CVB5-039cn	Aseptic meningitis	F	0	Jun. 2009	Stool	HQ685887	Coxsackievirus B5
Kor09-CVB5-050cn	Septicaemia	M	0	Jun. 2009	Stool	HQ685888	
Kor09-CVB5-056cn	Aseptic meningitis	F	11	Jul. 2009	Stool	HQ685889	
Kor09-CVB5-057cn	Aseptic meningitis	F	0	Jul. 2009	Stool	HQ685890	
Kor09-CVB5-058cn	Aseptic meningitis	F	0	Jul. 2009	Stool	HQ685891	
Kor09-CVB5-059cn	Aseptic meningitis	F	0	Jul. 2009	CSF	HQ685892	
Kor09-CVB5-073cn	Bronchopneumonia	F	2	Jul. 2009	Stool	HQ685893	
Kor09-CVB5-080cn	Aseptic meningitis	M	5	Jul. 2009	Stool	HQ685894	
Kor09-CVB5-081cn	Aseptic meningitis	F	0	Jul. 2009	Stool	HQ685895	

### Nucleotide sequencing and molecular typing

PCR products were purified using the QIA quick PCR purification kit (Qiagen, Valencia, CA). Purified DNA was added in a reaction mixture containing 2 μL of Big Dye terminator reaction mix (Applied Biosystems, Foster City, CA) and 2 pmoles of AN88 and AN89 primers (Table 1). Sequencing reactions were subjected to initial denaturation at 96°C for 1 min and 25 cycles consisting of 96°C for 10 sec, 50°C for 5 sec, and 60°C for 4 min in a Gene Amp PCR system 2700 (Applied Biosystems). The products were purified by precipitation with 100% cold ethanol and 3 M sodium-acetate (pH 5.8), and then loaded on a model 3100 automated genetic analyzer (Applied Biosystems). The molecular type of each isolates was determined by the serotype of the highest scoring strain in Genbank using the Basic Local Alignment Search Tool (BLAST); that is, the sequence of the enterovirus strain that gave the highest nucleotide similarity value with the query sequence [26].

### Sequence analysis of CVB 1 and CVB 5

Seventeen Korean CVB 1 and eight Korean CVB 5 strains that displayed a more informed profile were selected for sequence analysis (Table 1). Nucleotide and deduced amino acid sequences of candidate enterovirus isolates were compared with the reference strains using CLUSTAL W (version 1.81) and Megalign (DNASTAR) [27]. These programs are applied Needleman and Wunsch algorithm (M-NW similarity test). A similarity score between each pair of sequences was obtained manually after sequential pairwise alignment (M-NW similarity test) was performed. The quality of the alignment could not be measured, and the gap opening penalty (GOP) and the gap extension penalty (GEP) values were used as default values [28,29]. The phylogenetic relationships among the VP1 sequences of each virus isolate were inferred by using MEGA software v. 4.0. Maximum Composite Likelihood was used as the substitution method, while the neighbor-joining method was used to reconstruct the phylogenetic tree [30]. The reliability of the phylogenetic tree was determined by bootstrap re-sampling of 1,000 replicates.

### Nucleotide sequence accession numbers

The enterovirus candidates sequences reported here were deposited in the Genbank sequence database, with the accession numbers summarized in Table 1.

## Results

### Enterovirus detection and molecular typing

In 2008 and 2009, 1214 samples obtained from patients with aseptic meningitis and enterovirus-related disease were subjected to a diagnostic RT-PCR with cell culture that generated 436 bp amplicons, corresponding to a highly conserved domain in 5' NCR. Eighty two enteroviruses were isolated from 435 cases (18.9%) in 2008 and 107 enteroviruses were isolated from 779 cases (13.7%) in 2009. For molecular typing and phylogenetic analysis, the VP1 amplicons generated in the semi-nested PCR were sequenced and were determined to correspond with a 372 bp VP1 region. Gapped BLAST analyses were carried out and each virus was assigned the type that gave the highest VP1 identity score. The types in the 189 isolates were identified as ECV 30 (n = 42, 22.2%), CVB 1 (n = 37, 19.6%), CVB 5 (n = 21, 11.1%), ECV 6 (n = 19, 10.1%), EV 71 (n = 12, 6.3%), CVA 2 (n = 11, 5.8%), CVA 16 (n = 9, 4.8%), CVA 4 (n = 6, 3.2%), CVA 5 (n = 3, 1.6%), ECV 5 (n = 3, 1.6%), ECV 11 (n = 3, 1.6%), CVB 3 (n = 2, 1.1%), ECV 9 (n = 2, 1.1%), and ECV 16 (n = 1, 0.5%). VP1 amplicons were not generated from 18 samples by semi-nested PCR. (Table 2)

**Table 2:** Number of enterovirus types isolated in Chungnam, Korea, 2008 and 2009

Type of enterovirus	2008		2009	
	
	Isolate numbers	Percentage of subtotal	Isolate numbers	Percentage of subtotal
Coxsakievirus A2	-	-	11	10.3
Coxsakievirus A4	2	2.4	4	3.7
Coxsakievirus A5	-	-	3	2.8
Coxsakievirus A16	1	1.2	8	7.5
Coxsakievirus B1	3	3.7	34	31.8
Coxsakievirus B3	2	2.4	-	-
Coxsakievirus B5	-	-	21	19.6
Echovirus 6	19	23.2	-	-
Echovirus 7	3	3.7	-	-
Echovirus 9	2	2.4	-	-
Echovirus 11	1	1.2	2	1.9
Echovirus 16	1	1.2	-	-
Echovirus 30	42	51.2	-	-
Enterovirus 71	-	-	12	11.2
Untypable	6	7.3	12	11.2

Total	82	100	107	100

### Epidemiological features of enterovirus in Chungnam

**Figure 1:**
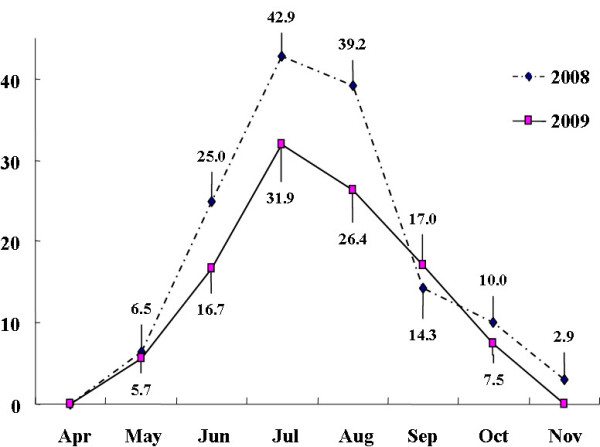
**Temporal distribution of enterovirus-positive cases in Chungnam, Korea, 2008 and 2009**.

Temporal distribution of the epidemic enterovirus in Chungnam was seasonal, with most cases occurring during the summer from June to September. The enterovirus detection rate in these months in 2008 and 2009, respectively, was 25.0% and 16.7% in June, 42.9% and 31.9% in July, 39.2% and 26.4% in August, and 14.3% and 17.0% in September (Figure 1).

**Figure 2:**
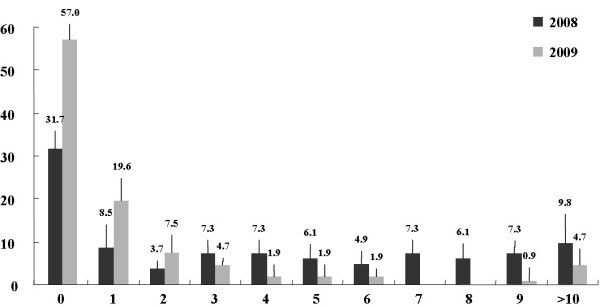
**Age distribution of enterovirus-positive patients in Chungnam, Korea, 2008 and 2009**.

Seventy four cases (90.2%) of 82 total samples in 2008 and 102 cases (95.3%) of 107 total cases in 2009 involved individuals < 10-years-of-age. Eight cases (9.8%) in 2008 and five cases (4.7%) in 2009 involved individuals > 10-years-of-age. The highest rate of enterovirus-positive samples were from patients < 1-year-of-age (31.7% in 2008 and 57.0% in 2009) (Figure 2.). Of the total 189 isolates, 121 were from males and 68 were from females, giving a male-to-female ratio of approximately 1.78:1.

The major clinical symptoms of hospitalized patients infected by enterovirus are summarized in Table 3. Aseptic meningitis was the major clinical manifestation (80.5% in 2008 and 56.1% in 2009). The other clinical symptoms were respiratory illness (6.1% in 2008 and 15.0% in 2009), acute gastroenteritis (4.9% in 2008 and 13.1% in 2009), herpangina (1.2% in 2008 and 7.5% in 2009), or hand-foot-mouth disease (1.2% in 2008 and 0.9% in 2009).

**Table 3:** Distribution of clinical symptoms for enterovirus-positive cases in Chungnam, Korea, 2008 and 2009

Clinical symptoms	2008		2009	
	
	Isolate numbers	Percentage of subtotal	Isolate numbers	Percentage of subtotal
Aseptic meningitis	66	80.5	60	56.1
Respiratory illness	5	6.1	16	15.0
Acute gastroenteritis	4	4.9	14	13.1
Herpangina	1	1.2	8	7.5
Hand foot mouth disease	1	1.2	1	0.9
Others	5	6.1	8	7.5
Total	82	100	107	100

### Sequence analysis of CVB 1 and CVB 5

**Figure 3:**
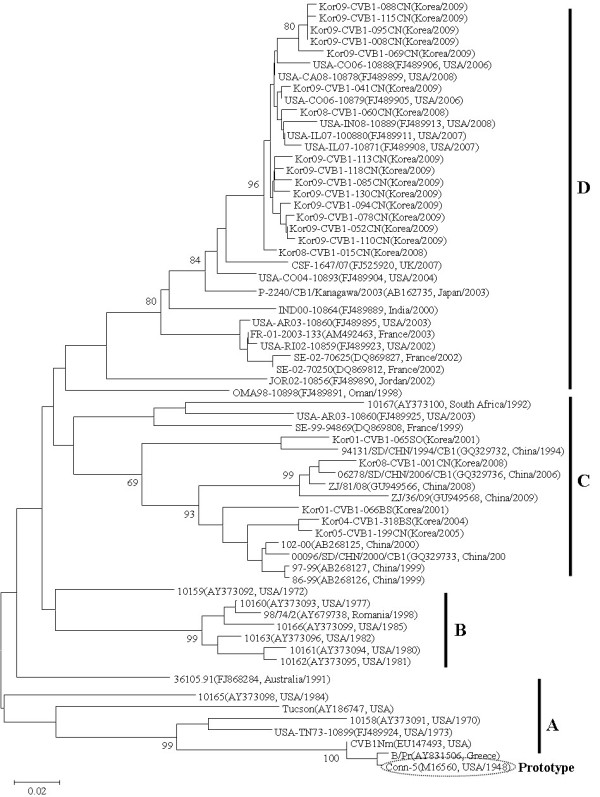
**Phylogenetic analysis based on a 270 bp sequence of the VP1 of CVB1 strains**. Nucleotide sequences were analyzed by the neighbor-joining method. The numbers at the branches indicate the bootstrap values for 1,000 replicates.

To analyze the genetic characteristics of CVB 1, the 21 CVB 1 isolates were examined by RT-PCR amplifying the VP1 region and sequencing. VP1 sequences for the CVB 1 isolates were compared with 43 foreign strains. The CVB 1 sequences segregated into four distinct clusters (A, B, C, and D) with some temporal and regional sub-clustering (Figure 3). Cluster A showed 15.7-29.2%, 19.3-32.1%, and 17.1-34.0% nucleotide divergence from cluster B, C, and D, respectively. Cluster B displayed 17.7-26.4% and 15.9-24.8% nucleotide divergence from cluster C and D, respectively. Cluster C shared 71.7-83.2% nucleotide identity with cluster D. All Korea strains (except for Kor08-CVB1-001CN) isolated from 2008 and 2009 belonged to cluster D.

**Figure 4:**
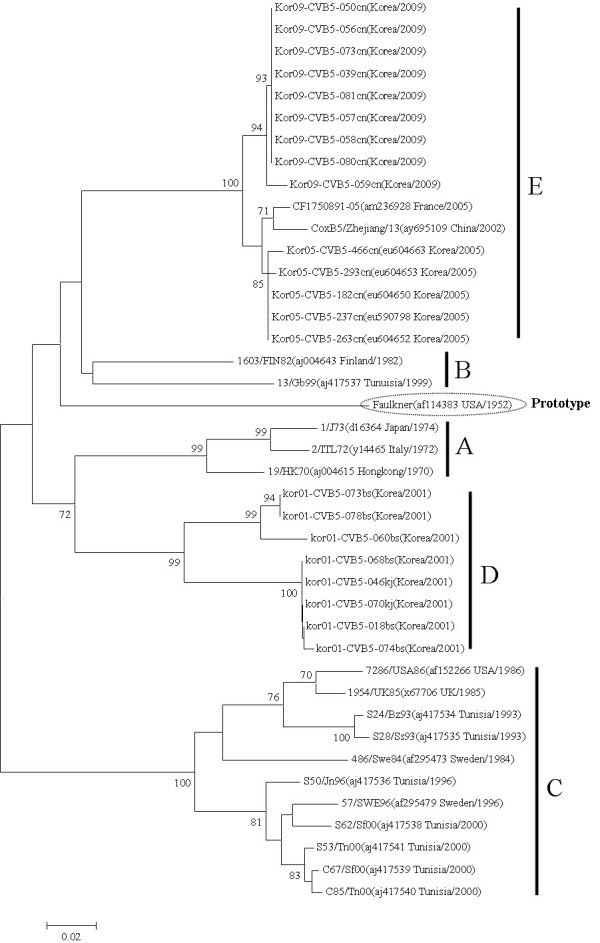
**Phylogenetic analysis based on a 302 bp sequence of the VP1 of CVB5 strains**. Nucleotide sequences were analyzed by the neighbor-joining method. The numbers at the branches indicate the bootstrap values for 1,000 replicates.

**Figure 5:**
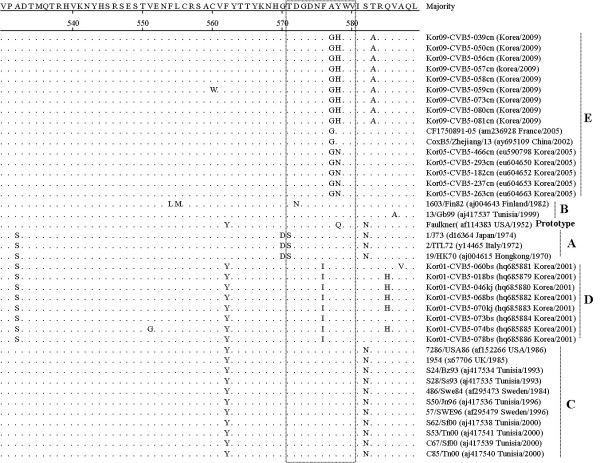
**Comparison of the deduced amino acid sequences of 41 CVB5 strains in the VP1 region**. The BC loop is boxed.

The VP1 sequences of 22 CVB 5 Korean isolates obtained in 2001, 2005, and 2009 were used to construct a phylogenetic tree with 19 reference strains from the GenBank database with the same serotype. The CVB 5 strains segregated in five distinct genetic groups supported by high bootstrap values (Figure 4). Cluster A showed 18.4-21.9%, 24.9-29.6%, 15.7-21.6%, and 17.5-22.3% nucleotide divergence from cluster B, C, D, and E, respectively. Cluster B showed 22.7-27.5% 21.1-23.6%, and 15.1-16.8% nucleotide divergence from cluster C, D, and E, respectively. Cluster C showed 22.1-29.3% and 22.1-27.4% nucleotide divergence from cluster D and E, respectively. Cluster D showed 19.4-22.5% nucleotide divergence with cluster E. The 2001 Korean isolates in belonged to cluster D, whereas the Korean isolates from 2005 and 2009 belonged to cluster E. Based on the amino acid sequence comparison of the VP1 region for isolates of the CVB 5 serotype, the 2009 Korean isolates were substituted at the positions 583 (T583A). AY was substituted for GH at positions 577 and 578 in the BC loop, as shown in Figure 5.

## Discussion

Various enterovirus serotypes are spreading globally by temporal and regional factors. Widely distributed throughout the year in tropical and semitropical regions, they are prominent in summer and fall, and far less detected in winter and spring months [31]. We previously reported the epidemic occurrence of enteroviruses isolated in Chungnam, Korea in 2005 and 2006 [11]. The current study extended these findings by investigating the difference of the epidemic pattern in 2008 and 2009. The data from 2008 and 2009 revealed a similar seasonal distribution in prevalence as found in 2005 and 2006. In both 2008 and 2009, enterovirus infections were most prevalent in July 42.9% and 31.9%, respectively). The highest rate of enterovirus-positive samples occurred in children < 1-year-old. However, differences with time were evident concerning the most prevalent enterovirus serotype: ECV 18 in 2005, ECV 5 in 2006, ECV 30 in 2008, and CVB 1 in 2009.

In the latest study, enteroviruses were most often isolated from patients with aseptic meningitis (80.5% in 2008 and 56.1% in 2009). Since the symptoms of enterovirus infection are quite variable, surveillance for other symptoms of enterovirus infection including aseptic meningitis could be prudent. The investigative scope for symptoms was extended in 2009; this may have reflected the marked 24% decrease in meningitis-positive samples.

PCR amplification followed by sequencing the VP1 region of the genome has been more recently used to type enteroviruses. Different VP1 sites have been targeted and demonstrated to contain serotype specific information [21,22]. Phylogenetic analysis revealed Korean ECV 6 and ECV 30 as the prevalent enterovirus types in 2008 [32,33]. Presently, a similar phylogenetic analysis revealed CVB 1 and 5 as the prevalent enterovirus types in Korea in 2009. Clustering or genotyping in combination with phylogenetic analysis is able to discriminate between lineages within a serotype and to identify emergent new variants or serotypes [24]. The sequences of the Korean CVB 1 isolates could be divided into four genetic clusters (A, B, C, and D) with at least 15% diversity between clusters according to PV genotypes [34]. Almost all the Korean CVB 1 isolates in 2008 and 2009 were in cluster D, with only one, "Kor08-CVB1-001CN", resident elsewhere, in cluster C. It is conceivable that "Kor08-CVB1-015CN" and "Kor08-CVB1-060CN" isolates were the origins of the 2009 Korean strains. The oldest CVB 1 isolates grouped in cluster A were viruses isolated from 1948 to the early 1980s. The viruses in clusters B and C were isolated from the 1970s to the mid-1980s and from the mid1990s to the present, respectively. The latest CVB 1 isolates clustered in group D. It seems appropriate to suggest that the grouping of these CVB 1 viruses may differ with time.

The VP1 region containing the BC loop is one of the main exposed regions of the viral capsid and has been suggested to include a serotype-specific antigenic neutralization [22,23]. We found amino acid sequence substitutions at nine amino acid sequences (532, 562, 570, 571, 576-578, 582, 583, and 585). Especially, the only The 2009 Korean isolates displayed a T→A replacement of only amino acid 583.

In the reconstruction of the phylogenetic tree based on the VP1 nucleotide sequences of Korean isolates and the reference strains of CVB 5, all Korean isolates recovered in 2001 were segregated from the other lineage groups. In cluster E, the 2009 Korean isolates were divided to the 2005 Korean isolates because of amino acid sequence substitutions at the amino acids 578 (N→H) and 583 (T→A). Consequently, we determined the epidemiological patterns of patients with enterovirus-related diseases in Korea in 2008 and 2009. Also, the most prevalent types in 2009 (Korean CVB 1 and CVB 5) genetically characterized using amino acid sequence comparison and phylogenetic analysis.

## Competing interests

The authors declare that they have no competing interests.

## Authors' contributions

BKA, YSG, PKS, HSY, and SJH performed molecular diagnosis and sequence analysis. LBH, YJS, RIS, KJK, and CYJ contributed to collection specimen and clinical diagnosis. CDS and PJS designed the study and critically revised the manuscript. All of the authors read and approved the final version of the manuscript.
